# Immune thrombocytopenic purpura increased risk of subsequent pancreatitis: A Nationwide population cohort study

**DOI:** 10.1038/s41598-019-53165-7

**Published:** 2019-11-15

**Authors:** Shih-Chi Wu, Sheng-fung Lin, Chu-Wen Fang, I-Ju Tsai, Wen-Chi Yang

**Affiliations:** 1 Graduate Institute of Clinical Medical Science, China Medical University, Taichung, Taiwan; 2 Trauma and Emergency Center, China Medical University Hospital, Taichung, Taiwan; 3 Division of Hematology and Medical Oncology, Department of Internal Medicine, E-DA Hospital, Kaohsiung, Taiwan; 4 Division of urology, Department of Surgery, Chi Mei Medical Center, Tainan, Taiwan; 5 Management Office for Health Data, China Medical University and Hospital, Taichung, Taiwan; 6 College of Medicine, China Medical University, Taichung, Taiwan; 7 Faculty of School of Medicine, College of Medicine, I-Shou University, Kaohsiung, Taiwan

**Keywords:** Experimental models of disease, Medical research

## Abstract

Immune thrombocytopenic purpura (ITP) is characterized by thrombocytopenia and bleeding diathesis. Pancreatitis is a very rare complication but may be fatal. We analyzed data of newly diagnosed ITP patients, excluding those with a history of splenectomy, unknown sex or date of birth, or preexisting pancreatitis at the time of ITP diagnosis, and compared these with selected age-, gender-, and index-year-matched controls, using the Taiwan National Health Insurance Research Database from 1996 to 2013. The study enrolled 100,177 ITP patients and 100,177 controls. We found that pancreatitis risk was higher in secondary ITP patients, regardless of age group, gender, baseline Charlson comorbidity index (CCI) score, history of biliary stone, hyperlipidemia, or alcoholism, than in the control population. Primary ITP patients with CCI score 1 and without biliary tract stone history also showed a higher pancreatitis risk than the controls. The incidence rate and cumulative incidence of pancreatitis were increased in primary, secondary, and unspecified ITP cases. These phenomena may be related to the presence of autoantibodies against glycoprotein IIb/IIIa, or to IgG4, microparticle obstruction, or sclerosis. We noted a direct association between ITP and the development of pancreatitis in Taiwan population.

## Introduction

Immune thrombocytopenic purpura (ITP) is an autoimmune disorder characterized by a low platelet count and mucocutaneous bleeding^1^. It is commonly assumed that ITP results from the presence of autoantibodies that cause accelerated platelet destruction or inhibit platelet production^2^. Based on a cut-off duration of 6 m, ITP is traditionally divided into acute and chronic forms^3^. In adults, ITP is primarily chronic, with higher prevalence in women than in men^3^. The prevalence of ITP is 20.3 per 100,000 persons in the United States of America (USA) and 50.29 per 100,000 in the United Kingdom (UK)^4,5^. The incidence of ITP ranges from 2.20 to 3.9 per 100,000 person-years from population based studies in France^6^, UK^7,8^, and Japan^9^.

There are few reports on ITP-specific comorbidities. A 14-y, population-based case control study in the UK found that ITP is associated with various medical conditions, and that some only occurred after onset of ITP; these included oral, infectious, gastrointestinal, and autoimmune disorders^10^. Immune pancreatitis complicated by ITP has been reported. The majority of these reported patients were Japanese individuals^11–20^. In our daily practice, we observed that few chronic ITP cases showed higher rates to get pancreatitis.

Acute pancreatitis patients demonstrate increased platelet adhesiveness and aggregation, resulting from an increase in platelet activation factors^21^ such as serotonin^22^, in combination with membrane integrin glycoprotein IIb/IIIa, membrane receptors for fibrinogen, and the calcium-dependent linkage between activated receptors and bivalent fibrinogen. These factors induce platelet aggregation, and are associated with proaggregatory mediators such as adenosine diphosphate (ADP), adenosine triphosphate (ATP), platelet factor 4, β-thromboglobulin, thromoboxane A2, fibrinogen, and thrombospondin^21^. However, we found no reports of an increase in the acute pancreatitis rate in association with ITP. Accordingly, this large-scale study in the Taiwanese population investigated the association between pancreatitis and ITP.

## Results

### Patient characteristics

**Figure 1:**
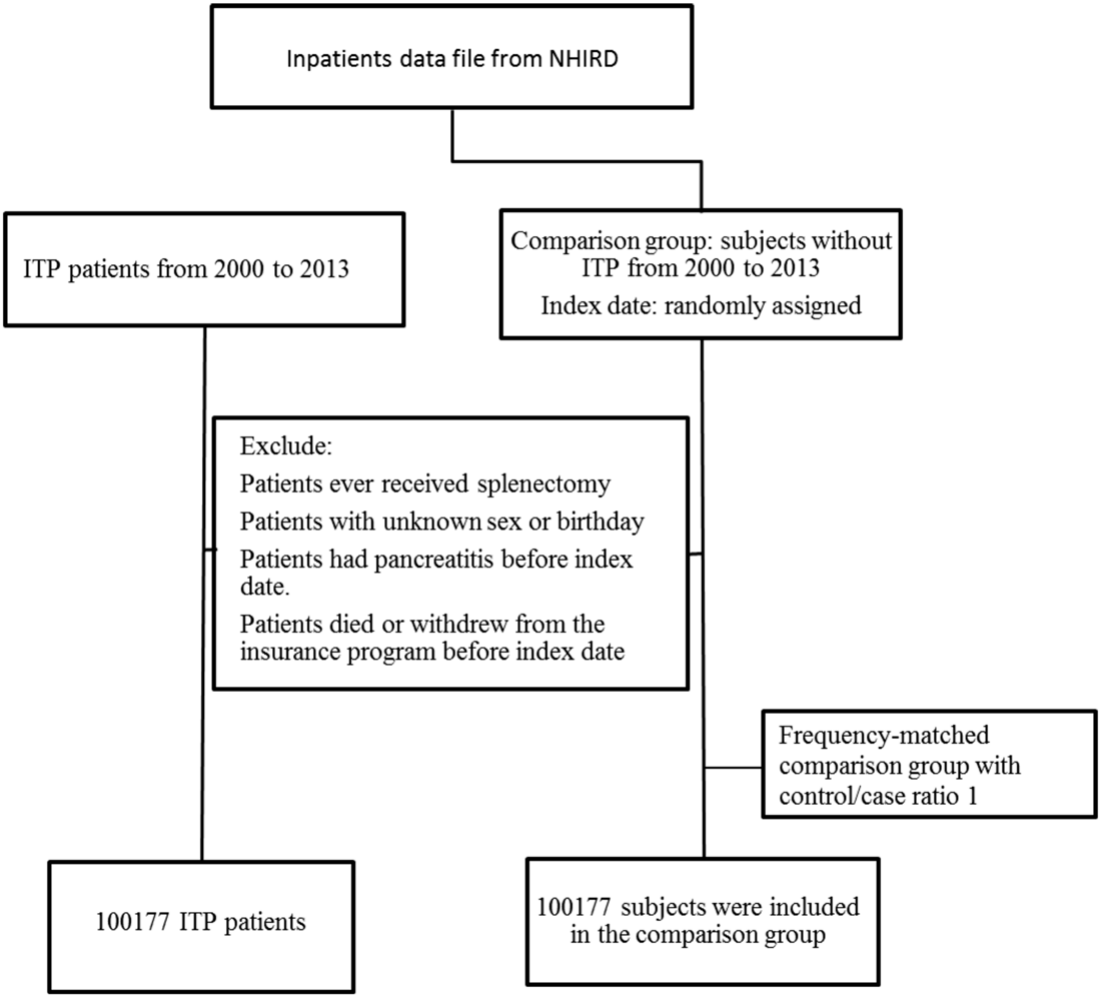
Flow chart of enrollment criteria.

The schema of this study is shown in Fig. 1. The mean age of both ITP and control groups was 60.3 years with majority subjects older than 45 y (Table 1). There were more men than women (56.7% vs. 43.3%) in both groups. ITP patients had higher percentages of biliary stone (4.9% vs. 2.2%) and alcoholism (4.6% vs. 0.4%) than the controls. The distributions of CCI score between the two groups were quite different. Only 33.4% of ITP patients had CCI score of 0, while 77.2% of the control group did.

**Table 1:** Baseline characteristics of ITP patients and comparison group.

	ITP	Comparison group	p-value
	(n = 100177)	(n = 100177)	
**Age, years**			
Mean (SD)	60.3 (17.7)	60.3 (17.7)	1
<45	20430 (20.4)	20430 (20.4)	
45–64	34584 (34.5)	34584 (34.5)	
≥65	45163 (45.1)	45163 (45.1)	
**Gender, n (%)**			
Female	43347 (43.3)	43347 (43.3)	1
Male	56830 (56.7)	56830 (56.7)	
**Baseline CCI**			
0	33506 (33.4)	77357 (77.2)	<0.0001
1	18440 (18.4)	10847 (10.8)	
2	20445 (20.4)	5998 (6.0)	
3+	27786 (27.7)	5975 (6.0)	
**Comorbidity, n (%)**			
Biliary stone	4928 (4.9)	2176 (2.2)	<0.0001
Hyperlipidemia	2612 (2.6)	2859 (2.9)	0.0007
Alcoholism	4583 (4.6)	413 (0.4)	<0.0001

ITP: immune thrombocytopenia purpura; CCI: Charlson comorbidity index.

### ITP increases pancreatitis risk

We evaluated the risk of pancreatitis in different stratifications in Tables 2, 3 and 4. Disease diagnoses were identified using the International Classification of Diseases, 9^th^ Revision, Clinical Modification (ICD-9-CM). We divided ITP patients into 3 subgroups according to the ICD-9-CM codes: primary (ICD9 code 287.3), secondary (ICD9 code 287.4), unspecified (ICD9 code 287.5). Overall ITP patients had significant higher risk of pancreatitis than the control group (adjusted HR = 1.22 with *P* = *0.0154* in primary ITP, Table 2; adjusted HR = 1.86 with *P* < *0.0001* in secondary ITP, Table 3; adjusted HR = 2.00 with *P* < *0.0001* in thrombocytopenia, unspecify, Table 4). When stratifying all patients by age, gender, and CCI score, secondary ITP patients and thrombocytopenia, unspecific patients had significant higher risks of pancreatitis than the control group in all stratifications. Primary ITP patients with baseline CCI score 1 showed a higher risk of pancreatitis. Among subjects without biliary stone, hyperlipidemia or alcoholism, ITP patients had significantly higher risk of pancreatitis than the controls. However, it showed the reverse result among subjects with biliary stone in unspecific thrombocytopenia patients (adjusted HR = 0.79 with 95% CI = 0.67–0.93, Table 4). A trend of lower pancreatitis risk in all three groups with alcoholism was also noted.

**Table 2:** Risk of pancreatitis in ITP patients compared with the comparison group^@^.

	ITP (Primary)			Comparison			HR (95%CI)		*Interact P*
	Event	Person-years	Incidence^†^	Event	Person-years	Incidence^†^	Crude	Adjusted	
Overall	60	29352	2.04	750	571246	1.31	1.56 (1.20, 2.02)**	1.22 (0.93, 1.59)	
Age									*0.0154*
<45	22	13104	1.68	73	134777	0.54	3.08 (1.91, 4.95)***	1.46 (0.87, 2.47)	
45–64	15	8351	1.80	193	212444	0.91	1.97 (1.16, 3.33)*	1.23 (0.72, 2.10)	
≥65	23	7897	2.91	484	224025	2.16	1.35 (0.89, 2.05)	0.89 (0.58, 1.35)	
Gender, n(%)									*0.0457*
Female	32	20445	1.57	303	252339	1.20	1.30 (0.91, 1.88)	1.14 (0.79, 1.66)	
Male	28	8907	3.14	447	318907	1.40	2.23 (1.52, 3.26)***	1.28 (0.87, 1.89)	
Baseline CCI									*0.7079*
0	22	20986	1.05	453	468972	0.97	1.08 (0.71, 1.66)	0.94 (0.61, 1.46)	
1	20	4464	4.48	132	55580	2.37	1.90 (1.19, 3.04)**	1.66 (1.01, 2.73)*	
2	7	2228	3.14	70	25846	2.71	1.16 (0.53, 2.51)	0.91 (0.41, 2.02)	
3+	11	1674	6.57	95	20848	4.56	1.45 (0.78, 2.71)	1.43 (0.75, 2.72)	
**Comorbidity, n(%)**									
Biliary stone									*0.0025*
No	39	27942	1.40	451	556275	0.81	1.71 (1.24, 2.38)**	1.58 (1.13, 2.21)**	
Yes	21	1410	14.9	299	14971	20.0	0.74 (0.47, 1.15)	0.75 (0.48, 1.18)	
Hyperlipidemia									*0.5466*
No	55	28034	1.96	680	549126	1.24	1.58 (1.20, 2.08)**	1.24 (0.94, 1.64)	
Yes	5	1318	3.79	70	22120	3.16	1.17 (0.47, 2.91)	0.88 (0.34, 2.26)	
Alcoholism									*0.3864*
No	52	28937	1.80	699	568482	1.23	1.46 (1.10, 1.94)**	1.23 (0.93, 1.64)	
Yes	8	416	19.3	51	2763	18.5	1.03 (0.49, 2.17)	0.80 (0.37, 1.74)	

^†^Per 1000 person-years.

*p < 0.05; **p < 0.01; ***p < 0.001.

^@^Stratified by age, sex, and baseline characteristics.

Variables listed in Table 1 were considered as potential risk factors and were all put in the adjusted model.

**Table 3:** Risk of pancreatitis in ITP patients compared with the comparison group^@^.

	ITP (Secondary)			Comparison			HR (95%CI)		*Interact P*
	Event	Person-years	Incidence^†^	Event	Person-years	Incidence^†^	Crude	Adjusted	
Overall	101	16777	6.02	750	571246	1.31	4.41 (3.57, 5.44)***	1.86 (1.46, 2.37)***	
Age									<*0.0001*
<45	28	4032	6.94	73	134777	0.54	12.0 (7.69, 18.7)***	2.17 (1.17, 4.02)*	
45–64	45	6634	6.78	193	212444	0.91	7.17 (5.14, 10.0)***	2.10 (1.36, 3.23)***	
≥65	28	6111	4.58	484	224025	2.16	2.13 (1.45, 3.12)***	1.34 (0.90, 1.99)	
Gender, n(%)									*0.0003*
Female	21	7350	2.86	303	252339	1.20	2.34 (1.50, 3.65)***	1.28 (0.81, 2.05)	
Male	80	9427	8.49	447	318907	1.40	5.74 (4.51, 7.32)***	2.16 (1.62, 2.89)***	
Baseline CCI									*0.2022*
0	15	5289	2.84	453	468972	0.97	2.94 (1.76, 4.92)***	1.77 (1.05, 2.98)*	
1	18	3712	4.85	132	55580	2.37	1.97 (1.20, 3.24)**	1.70 (0.99, 2.93)	
2	21	3540	5.93	70	25846	2.71	2.02 (1.23, 3.31)**	1.77 (1.01, 3.09)*	
3+	47	4236	11.10	95	20848	4.56	2.53 (1.78, 3.62)***	1.84 (1.21, 2.81)**	
**Comorbidity, n(%)**									
Biliary stone									<*0.0001*
No	69	15925	4.33	451	556275	0.81	5.11 (3.95, 6.61)***	2.20 (1.64, 2.95)***	
Yes	32	852	37.5	299	14971	20.0	1.70 (1.18, 2.45)**	1.48 (0.99, 2.21)	
Hyperlipidemia									*0.7038*
No	92	16239	5.67	680	549126	1.24	4.39 (3.52, 5.47)***	1.87 (1.45, 2.41)***	
Yes	9	539	16.71	70	22120	3.16	4.76 (2.37, 9.57)***	2.22 (1.01, 4.89)*	
Alcoholism									<*0.0001*
No	82	15920	5.15	699	568482	1.23	4.02 (3.19, 5.06)***	2.40 (1.88, 3.06)***	
Yes	19	857	22.2	51	2763	18.5	1.10 (0.63, 1.91)	0.62 (0.32, 1.19)	

^†^Per 1000 person-years.

*p < 0.05; **p < 0.01; ***p < 0.001.

^@^Stratified by age, sex, and baseline characteristics.

Variables listed in Table 1 were considered as potential risk factors and were all put in the adjusted model.

**Table 4:** Risk of pancreatitis in ITP patients compared with the comparison group^@^.

	Thrombocytopenia, unspecify			Comparison			HR (95%CI)		*Interact P*
	Event	Person-years	Incidence^†^	Event	Person-years	Incidence^†^	Crude	Adjusted	
Overall	1552	287435	5.40	750	571246	1.31	3.83 (3.51, 4.18)***	2.00 (1.81, 2.21)***	
Age									<*0.0001*
<45	489	80627	6.06	73	134777	0.54	10.4 (8.12, 13.3)***	4.65 (3.56, 6.07)***	
45–64	581	104313	5.57	193	212444	0.91	5.63 (4.78, 6.63)***	2.67 (2.22, 3.21)***	
≥65	482	102495	4.70	484	224025	2.16	2.10 (1.85, 2.39)***	1.29 (1.12, 1.48)***	
Gender, n(%)									<*0.0001*
Female	489	129527	3.78	303	252339	1.20	2.99 (2.59, 3.46)***	1.71 (1.46, 2.00)***	
Male	1063	157908	6.73	447	318907	1.40	4.42 (3.95, 4.94)***	2.20 (1.94, 2.49)***	
Baseline CCI									<*0.0001*
0	356	134236	2.65	453	468972	0.97	2.70 (2.35, 3.11)***	1.45 (1.24, 1.69)***	
1	377	59155	6.37	132	55580	2.37	2.59 (2.12, 3.15)***	1.63 (1.31, 2.02)***	
2	329	44338	7.42	70	25846	2.71	2.61 (2.01, 3.37)***	1.79 (1.36, 2.35)***	
3+	490	49707	9.86	95	20848	4.56	2.05 (1.65, 2.56)***	1.35 (1.06, 1.70)*	
**Comorbidity, n(%)**									
Biliary stone									<*0.0001*
No	1132	266946	4.24	451	556275	0.81	4.80 (4.30, 5.36)***	2.85 (2.53, 3.22)***	
Yes	420	20489	20.5	299	14971	20.0	0.96 (0.82, 1.11)	0.79 (0.67, 0.93)**	
Hyperlipidemia									*0.0248*
No	1433	274558	5.22	680	549126	1.24	3.92 (3.58, 4.30)***	2.05 (1.85, 2.27)***	
Yes	119	12877	9.24	70	22120	3.16	2.71 (2.02, 3.64)***	1.32 (0.94, 1.85)	
Alcoholism									<*0.0001*
No	1249	271351	4.60	699	568482	1.23	3.49 (3.18, 3.83)***	2.05 (1.85, 2.26)***	
Yes	303	16084	18.8	51	2763	18.5	0.90 (0.67, 1.22)	0.74 (0.54, 1.00)	

†Per 1000 person-years.

*p < 0.05;**p < 0.01; ***p < 0.001.

^@^Stratified by age, sex, and baseline characteristics.

We also compared the risk of pancreatitis among all subgroups with that of the control group. All ITP subgroups had significant higher risk of pancreatitis. Among ITP group, primary ITP patients had lower risk of pancreatitis than unspecified ITP patients (adjusted HR = 0.62 with 95% CI = 0.48–0.81. Table 5).

**Table 5:** Risk of pancreatitis in ITP subgroups compared with the comparison group.

	Event	Person-years	Incidence^†^	Adjusted HR (95%CI)	
Comparison	750	571246	1.31	1 (reference)	
ITP					
Primary	60	29352	2.04	1.32 (1.01, 1.71)*	0.62 (0.48, 0.81)***
Secondary	101	16777	6.02	2.07 (1.67, 2.56)***	0.99 (0.81, 1.21)
Unspecified	1552	287435	5.4	1.99 (1.8, 2.19)***	1 (reference)

^†^Per 1000 person-years.

*p < 0.05; **p < 0.01; ***p < 0.001.

### ITP increases pancreatitis risk with time

**Figure 2:**
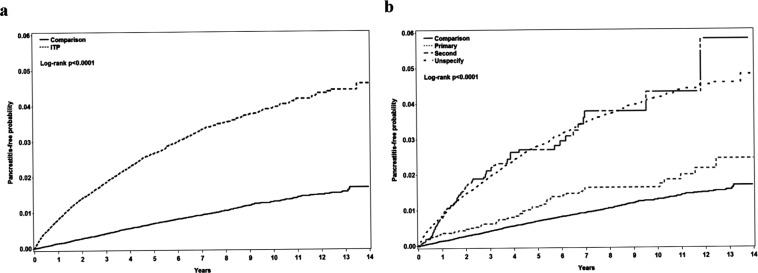
Cumulative incidence of pancreatitis in (**a**). ITP patients and the control group; (**b**). ITP subgroups, including primary, secondary, and unspecified groups, and the control group. ITP: immune thrombocytopenia purpura.

We analyzed the cumulative incidence of pancreatitis in ITP group, ITP subgroups and the control group. There was a significantly higher incidence of pancreatitis in ITP group/ITP subgroups, especial in secondary and unspecified groups, than in the control group (Fig. 2, Log-rank p < 0.0001).

## Discussion

Idiopathic thrombocytopenic purpura (ITP) is an acquired, immune-mediated disease of adults and children, with increasing of bleeding risk, that caused by transient or persistent decrease of the platelet count^23^. In addition, ITP patients were with increased susceptibility to bleeding because of antibody- and cell-mediated destruction of platelets and the suppression of platelet production. Moreover, the destruction and suppression of platelets are due to the autoimmune reactions of B cell (and sometimes CD8+ T cell) that directed against circulating platelets and megakaryocytes^1^.

ITP complicated by pancreatitis is very rare^10^. We used ICD-9 code including primary ITP, secondary ITP, and thrombocytopenia, unspecific to analysis if ITP increasing pancreatitis risk. The reason we chose those 3 codes is that we want to screen not only primary, but also secondary ITP effect on pancreatitis risk. We also pick up code 287.5, defined as thrombocytopenia, unspecific, because some clinician would use this broad define code, instead of more specific code, like 287.3 (primary ITP) or 287.4 (secondary ITP). Many patients putting in thrombocytopenia, unspecific item may be primary ITP ones, but less secondary ITP ones. Based on nationwide data in Taiwan, we found that the risk of pancreatitis increased in ITP patients, regardless of age, gender, and base line CCI. Primary ITP patients had higher pancreatitis risk in baseline CCI score 1 populations, but not in other CCI score ones. Biliary tract stones, hyperlipidemia, and alcoholism have been reported to increase pancreatitis risk^24–26^. In the Taiwanese population, primary ITP patients without biliary tract stones, and secondary ITP ones without biliary tract stones, hyperlipidemia, or alcoholism had a higher risk of pancreatitis than controls. A trend toward increasing pancreatitis risk was observed in ITP patients with hyperlipidemia, compared with that in controls, but the difference was only significant in secondary ITP group. However, primary ITP patients with a biliary stone history and alcoholism had a lower risk of pancreatitis than non-ITP patients. This suggests that biliary stones and alcoholism are stronger risk factors for pancreatitis than ITP. In addition, we aimed to determine whether the risk was different between primary and secondary ITP; the latter may be associated with other autoimmune diseases (eg, systemic lupus erythematosus [SLE], antiphospholipid antibody syndrome [APS], immune thyroid disease, or Evans syndrome), a lymphoproliferative disease (eg, chronic lymphocytic leukemia [CLL] or large granular T-lymphocyte lymphocytic leukemia), or chronic infection, eg, with *Helicobacter pylori*, human immunodeficiency virus (HIV), or hepatitis C virus (HCV)^27^. Our data showed that pancreatitis risk was higher in both primary and secondary ITP patients than in controls. However, the strength of association was less in the primary ITP group than in secondary or unspecified ITP groups. We may see stronger association in primary ITP group after expansion the collection patient number and years. In daily practice, we observed that ITP patients with obvious risk factors, including other autoimmune diseases, drugs, and infections, tended to develop secondary ITP. Most ITP patients without obvious risk factors were recorded as unspecified. The majority of unspecified ITP patients probably had primary ITP.

One possible cause of induced pancreatitis in ITP is splenectomy. Qu *et al*. reported a higher risk of pancreatitis in 604 post-splenectomy patients, possibly due to pancreatic injury or a fistula caused by surgery^28^. Therefore, in order to avoid this bias, we have excluded post-splenectomy patients in the current series. In addition, corticosteroids, the primary medication used in ITP, can also induce pancreatitis, and there were reported steroid-induced pancreatitis^29–31^. However, a meta-analysis and other reports showed a protective role of steroids for pancreatitis^32–34^. This inconsistency may indicate further investigations were needed for the role of steroid in this issue.

Other possible mechanisms of increased pancreatitis risk in ITP patients include antibody-associated pathways. One example is the IgG4-associated disease. Patients with autoimmune pancreatitis and ITP may share IgG4 antibodies. Patients with concurrent autoimmune pancreatitis and ITP reportedly had increased IgG4 levels^16,20^. In another mechanism, autoantibodies may target platelet membrane proteins. Autoantibodies against platelet glycoprotein (GP) IIb/IIIa and/or GPIb-IX are found in the majority of ITP patients, which could be detected using antigen-specific assays. In addition, multiple antibodies could be produced by many patients, which might be attributed to the phenomenon of epitope spreading. Once produced, autoantibodies may either bind to platelets or megakaryocytes, which cause destruction by either phagocytosis or complement activation and lysis in the former, and decreased thrombopoiesis in the latter^35^. In mouse studies, anti-GPIbα monoclonal antibodies induced severe and irreversible thrombocytopenia (<3% of normal) through Fc-independent mechanisms. Monoclonal antibodies against conformational epitopes in GPIIb/IIIa- or anti-GPIIb/IIIa-specific inflammatory reactions could induce irreversible thrombocytopenia, hematocrits decrease, hypothermia, acute systemic reactions, and a paradoxical loss of surface GPIIb/IIIa on platelets in *vivo*. The loss of surface GPIIb/IIIa implied the formation of platelet-derived microparticles^36^. These microparticles may obstruct small vessels and cause ischemic changes in terminal organs, including the pancreas, leading to pancreatitis. We analyzed pancreatitis risk after a diagnosis of ITP. We noted a time-dependent, higher pancreatitis incidence rate and cumulative incidence in all ITP patients, including primary, secondary, and unspecified groups, than in controls. The incidence of acute pancreatitis increased with age^37^. In our study, we showed higher accumulation incidence of pancreatitis in ITP patients. This indicates that increasing pancreatitis risk in ITP patients may be related to platelet-derived microparticles and the loss of platelet surface GPIIb/IIIa due to an anti-GPIIb/IIIa antibody effect.

The possible mechanism of developing pancreatitis in secondary ITP is more complex and may relate to underline disease. For examples, HCV infects pancreas acinar cells and pancreatic duct epithelial cells directly and leads to pancreatitis^38,39^. Immunosuppression in HIV infection patients is related to increase risk of pancreatitis^40^. Autoimmune disease like SLE related pancreatitis may induced by vascular damage (including vasculitis, intimal thickening, immune complex deposition, occlusion of arteries, and arterioles), autoantibody production, abnormal cellular immune response, and drug toxicity^41^. Leukemic cells infiltration in the pancreas in CLL patients, showed pancrease parenchymal destruction and fibrous scars, thereby resembling chronic pancreatitis^42^. And drug related pancreatitis, eg asparaginase^43^ for acute lymphoblastic leukemia and Highly Active Anti-Retroviral Therapy (HAART) for HIV infection^40^.

The limitation of this study describe below. In the NHIRD, the disease was defined based on the International Classification of Diseases, Ninth Revision, Clinical Modification code. The disease code(s) of the patients is according to the diagnosis of specialists. Therefore, in the current study, it is hard to obtain precise diagnosis of ITP due to databank limitation and only diagnosis codes were available. For example, only 4 numbers code for ITP in our database makes it difficult to identify congenital and hereditary thrombocytopenia purpura. But most likely, congenital disease can be excluded by age stratification. However, all the insurance claims in Taiwan were scrutinized and coded by medical reimbursement specialists and peer reviewed according to the standard diagnosed criteria. If these doctors or hospitals commit errors in diagnoses or coding, they will be punished with a lot of penalties. Thus, medical staffs were very concerned about the correct diagnosis codes, and we think the codes regarding diagnoses were highly reliable.

In conclusion, adult ITP patients, especially secondary ITP ones without biliary stones, hyperlipidemia, or history of alcoholism, have a higher risk of pancreatitis than controls. Primary adult ITP patients also show higher risk of pancreatitis, especial in patients without biliary stone history., than controls. That may be related to immune mechanisms.

## Patients and Methods

### Data source

This study used inpatient admissions data from the Taiwan National Health Insurance Research Database from 1996 to 2013. Established in 1995, the Taiwan National Health Insurance (NHI) program covers over 99% of the population and contracts with 93% of the medical institutions in Taiwan^44^. Disease diagnoses were identified using the International Classification of Diseases, 9^th^ Revision, Clinical Modification (ICD-9-CM). A disease diagnosis without valid supporting clinical findings may be considered a fraudulent claim by the NHI, with a penalty 100-fold greater than the payment claimed by the treating physician or hospital.

Patients aged 18 year-old or more, with newly diagnosed ITP (ICD-9 codes: 287.3–287.5) between 2000 and 2013, were identified. The date of ITP diagnosis was considered the study index date. Patients with the following criteria before the index date were excluded: (1) history of splenectomy; (2) unknown sex or birthday; (3) diagnosis of pancreatitis; or (4) deceased or withdrawn from the NHI program. We randomly assigned index dates for subjects who had never been diagnosed with ITP and selected age-, gender-, and index-year-matched subjects in our control group, using a control/case ratio of 1. We included 100,177 patients in the ITP group and 100,177 in the control group (Fig. 1). All patients were followed up from the index date to the first occurrence of one of the following: pancreatitis, withdrawal from the NHI program, or the last day of 2013. Baseline comorbidities, including biliary stones (ICD-9 code: 574), hyperlipidemia (ICD-9 code: 272), and alcoholism (ICD-9 codes: 291, 303, 305.00, 305.01, 305.02, 305.03, 571.0–571.3, 790.3, and V11.3) diagnosed before the index date were also considered. The Charlson comorbidity index (CCI) score was calculated for each subject.

The details of ICD-9 codes of ITP show as below.

287.3 Primary thrombocytopenia

−287.30 Primary thrombocytopenia, unspecified
−287.31 Immune thrombocytopenic purpura
−287.32 Evans’ syndrome
−287.33 Congenital and hereditary thrombocytopenic purpura
−287.39 Other primary thrombocytopenia
  287.4 Secondary thrombocytopenia
- 287.41 Posttransfusion purpura
- 287.49 Other secondary thrombocytopenia

287.5 Thrombocytopenia, unspecified

In our cohort, we defined primary ITP with ICD-9 codes of 287.30, 287.31 and 287.39, thrombocytopenia, unspecified with ICD-9 code of 287.5, and secondary ITP with ICD-9 codes other than previous two groups.

### Statistical analysis

Differences in baseline characteristics between ITP patients and the control group were examined using chi-square tests for categorical variables and the Wilcoxon rank-sum test for age. Hazard ratios (HRs) with 95% confidence intervals (95% CIs) were calculated using Cox regressions. Cumulative incidence curves were based on the Kaplan-Meier method, and the differences in cumulative incidence between groups were examined using the log-rank test. All statistical analyses were performed with SAS software version 9.4 (SAS Institute Inc., Carey, NC, USA). A two-tailed p-value below 0.05 was considered significant.

### Ethical Approval

This article does not contain any studies with human participants or animals performed by any of the authors.

### Disclosure

This study used the National Health Insurance Research Database established by the National Health Research Institutes, with the authorization of the Bureau of National Health Insurance, Ministry of Health and Welfare of Taiwan. The interpretations and the conclusions contained herein do not represent the opinion of the aforementioned agencies and institutions.
